# A new species of *Cuspidevia* Jäch & Boukal, 1995 (Coleoptera, Elmidae) from south China

**DOI:** 10.3897/BDJ.12.e117248

**Published:** 2024-03-21

**Authors:** Dongju Bian, Yuqi Hu, Yanfeng Tong

**Affiliations:** 1 Key Laboratory of Forest Ecology and Management, Institute of Applied Ecology, Chinese Academy of Sciences, Shenyang 110016, China Key Laboratory of Forest Ecology and Management, Institute of Applied Ecology, Chinese Academy of Sciences Shenyang 110016 China; 2 University of Chinese Academy of Sciences, Beijing 100049, China University of Chinese Academy of Sciences Beijing 100049 China; 3 Life Science College, Shenyang Normal University, Shenyang 110034, China Life Science College, Shenyang Normal University Shenyang 110034 China

**Keywords:** riffle beetles, *Cuspidevia*, new species, Guangdong, Guangxi, Jiangxi

## Abstract

**Background:**

*Cuspidevia* Jäch & Boukal, 1995 is a member of the tribe Macronychini which has the following features: antennae short, 6-10 segmented, aedeagus long and cylindrical, paramere very small, slender or lacking. This genus currently comprises only three species. All species are distributed in China.

**New information:**

*Cuspideviapilosus* sp. nov. is reported from Guangdong, Guangxi and Jiangxi Provinces in China. Habitus and diagnostic features of the new species are illustrated.

## Introduction

The genus *Cuspidevia* was established by Jäch and Boukal (1995). Only three species have been described, i.e. *Cuspideviabrevis* Bian & Ji, 2010, *C.jaechi* Bian & Ji, 2010 and *C.velaris* Jäch & Boukal, 1995 (Bian and Ji 2010). According to the original description of this genus, it has the following characteristics (see Jäch and Boukal (1995)): body elongate, scarcely punctate and pubescent, glabrous; antennae 8-segmented; anterior angles of pronotum strongly acuminately produced anteriad; usually with a short, shallow, longitudinal sulcus; elytral apices densely granulate, separately acuminately produced; elytral striae almost obsolete, with a granulate carina on the seventh interval.

In this paper, a new species of the genus *Cuspidevia*, collected from Guangdong, Guangxi and Jiangxi, China, is described and illustrated.

## Materials and methods

Specimens were examined with a Leica M205c stereomicroscope. Male genitalia were placed in concentrated lactic acid in a cavity slide for several hours before they were examined. Habitus and genitalia photographs were made with Keyence VHX-2000 Super Resolution Digital Microscope System. The first strial interval refers to the sutural interval. The type specimens of the new species were deposited in the Institute of Applied Ecology, Chinese Academy of Sciences, Shenyang, China (IAECAS) and the Institute of Entomology, Guizhou University, Guiyang, China (GUGC). Abbreviations used in the text: BL–body length = PL+EL, BW–maximum width of body (= EW), EL–elytral length, EW–maximum width of elytra, PL–pronotal length, PW–maximum width of pronotum.

## Taxon treatments

### Cuspidevia pilosus sp. nov.

88AD51C3-F2D9-5B3B-9D87-713184D19B82

1F35875B-8AF9-4790-915B-5EE146E479C2

#### Materials

- **Type status:** Holotype. **Occurrence:** recordedBy: Peng & Sun (39); individualCount: 1; sex: male; occurrenceID: 72C87AB3-955D-52D9-BA59-476DC72E9AC7; **Taxon:** scientificName: *Cuspideviapilosus*; order: Coleoptera; family: Elmidae; **Location:** country: China; stateProvince: Guangdong; county: Shaoguan, Ruyuan; locality: Nanling; verbatimElevation: 626 m; verbatimCoordinates: 113°3’ 2” E, 25°54’44” N; **Event:** eventDate: 27.11.2017; **Record Level:** institutionCode: IAECAS
- **Type status:** Paratype. **Occurrence:** recordedBy: Peng & Sun (39); individualCount: 7; sex: 4 males, 3 females; occurrenceID: C661F826-B3E7-5072-9626-028417C88998; **Taxon:** scientificName: *Cuspideviapilosus*; order: Coleoptera; family: Elmidae; **Location:** country: China; stateProvince: Guangdong; county: Shaoguan, Ruyuan; locality: Nanling; verbatimElevation: 626 m; verbatimCoordinates: 113°3’ 2” E, 25°54’44” N; **Event:** eventDate: 27.11.2017; **Record Level:** institutionCode: IAECAS
- **Type status:** Paratype. **Occurrence:** recordedBy: Peng & Sun (41); individualCount: 2; sex: male; occurrenceID: C64280C0-F287-54A1-9492-47205B9F5F8F; **Taxon:** scientificName: *Cuspideviapilosus*; order: Coleoptera; family: Elmidae; **Location:** country: China; stateProvince: Guangdong; county: Shaoguan, Ruyuan; locality: Nanling; verbatimElevation: 755 m; verbatimCoordinates: 113°2’ 45” E, 24°55’2” N; **Event:** eventDate: 27.11.2017; **Record Level:** institutionCode: IAECAS
- **Type status:** Paratype. **Occurrence:** recordedBy: Bian; individualCount: 1; sex: male; occurrenceID: 33457608-E5EB-5D95-A93A-CE107826D0A2; **Taxon:** scientificName: *Cuspideviapilosus*; order: Coleoptera; family: Elmidae; **Location:** country: China; stateProvince: Guangdong; county: Shaoguan, Shixing; locality: Chebaling Nat. Res.; verbatimElevation: 374 m; verbatimCoordinates: 114.250760° E, 24.719415° N; **Event:** eventDate: 06.08.2022; **Record Level:** institutionCode: IAECAS
- **Type status:** Paratype. **Occurrence:** recordedBy: Bian, Guo & Tong (9); individualCount: 5; sex: 2 males, 3 females; occurrenceID: 56FC4DFD-13A4-5E37-9538-183D8205501B; **Taxon:** scientificName: *Cuspideviapilosus*; order: Coleoptera; family: Elmidae; **Location:** country: China; stateProvince: Guangxi; county: Liuzhou, Rongshui; locality: Xishan Forest; **Event:** eventDate: 22.11.2011; **Record Level:** institutionCode: IAECAS
- **Type status:** Paratype. **Occurrence:** recordedBy: Bian, Guo & Tong (13); individualCount: 4; sex: male; occurrenceID: 09086976-8F61-519D-93D1-296B469DDBB4; **Taxon:** scientificName: *Cuspideviapilosus*; order: Coleoptera; family: Elmidae; **Location:** country: China; stateProvince: Guangxi; county: Guilin, Longsheng; locality: Huaping; **Event:** eventDate: 27.11.2011; **Record Level:** institutionCode: IAECAS
- **Type status:** Paratype. **Occurrence:** recordedBy: Zhen-Xing Ma; individualCount: 2; sex: male; occurrenceID: 55D1211F-2AB7-5020-870B-0648EB434E27; **Taxon:** scientificName: *Cuspideviapilosus*; order: Coleoptera; family: Elmidae; **Location:** country: China; stateProvince: Jiangxi; county: Longnan; locality: Jiulianshan N. R.; **Event:** eventDate: 19.09.2020; habitat: on stone of a stream; **Record Level:** institutionCode: GUGC

#### Description

**Figure 1.**
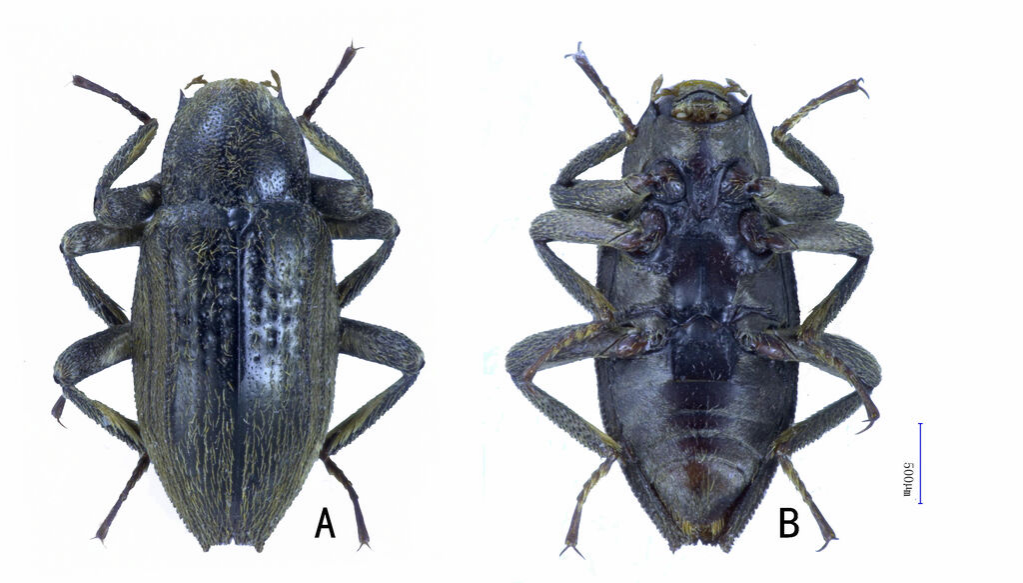
*Cuspideviapilosus* sp. nov., habitus. **A** dorsal view; **B** ventral view.

BL 3.0 mm, BW 1.3 mm. Habitus see Fig. 1A and B. Body elongated obovate, dorsal surface black, except anterior margin of pronotum yellowish-brown, ventral surface dark brown. Legs brown, tarsi slightly lighter, mouth and antennae yellowish-brown.

Head. Labrum transverse, basal half densely micro-reticulate, distal half smooth and shiny, sparsely punctate and densely pubescent, with long setae laterally. Clypeus and frons densely punctate and pubescent, with some granules.

**Figure 2.**
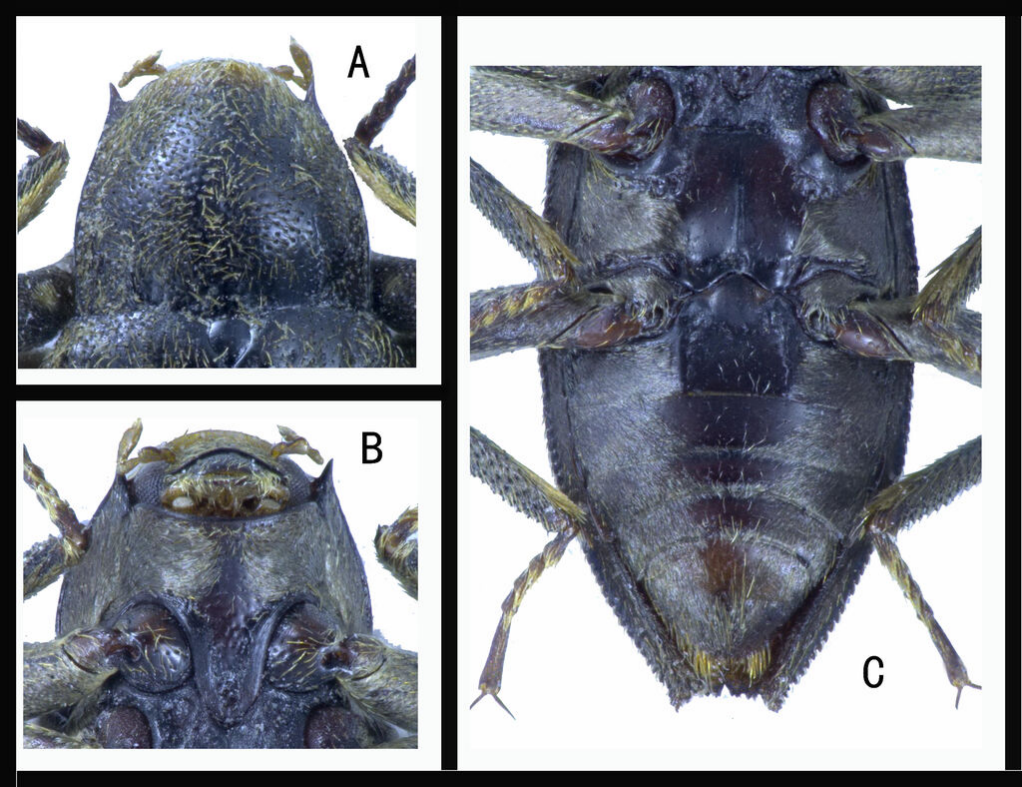
*Cuspideviapilosus* sp. nov., holotype**. A** pronotum; **B** prosternum and prosternal process; **C** mesoventrite, metaventrite and ventrites.

Pronotum. PL 0.85 mm, PW 0.90 mm. Pronotum (Fig. 2A) subparallel in basal 0.4, distinctly attenuated anteriorly. Anterior angles acute, distinctly produced, posterior angles almost right-angled. Punctures and yellow pubescence densely distribute. Median sulcus is absent. Base with two rows of granules (3–4 granules). Sublateral carinae present in basal 0.3, slightly elevated.

Elytra. Elytra broadest at the middle, slightly narrowed anteriorly and distinctly attenuated posteriorly. Striae not developed, the first striae extending from base to basal 0.4, with large punctures deeply impressed (separated by 1–1.5 diameters), the striae Ⅱ to Ⅳ extending from basal 0.2 to 0.4. Intervals smooth and shiny, small punctures sparsely distributed and yellowish pubescence is dense. Intervals Ⅱ to Ⅳ distinctly elevated in basal 0.2. Intervals V, Ⅶ and Ⅷ carinate. Carinae V and VII extending from basal 0.1 to apex, carinae VIII extending from basal 0.1 to distal 1/6. Plastron is present from intervals 5 to lateral margin. Each elytral apex with an apical projection.

Ventral side of thorax. Prosternum densely pubescent. Prosternal process (Fig. 2B) elongated, slightly narrowed from base to apex, apex broadly rounded, lateral margins distinctly rimmed; surface slightly wrinkled, punctures sparsely distributed, almost without pubescence. Metaventrite (Fig. 2C) distinctly impressed in posterior 0.6, disc almost without pubescence, micro-reticulate, with a few small punctures; lateral areas densely pubescent. Two rows of large punctures on each side, one is behind the mesocoxae and the other one in front of the metacoxae.

Ventrites I–V. Middle discs of ventrites I–IV and basal 0.2 disc of ventrite V smooth and shiny, only with a few short setae and small punctures, other areas of all ventrites densely pubescent. Ventrite I concave in basal half, with a pair of ad-median carinae. Apex of ventrite V not emarginate.

**Figure 3.**
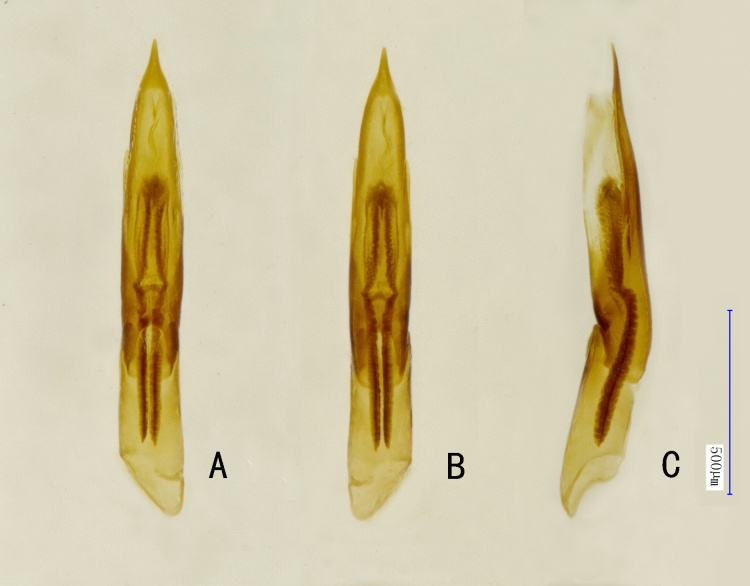
*Cuspideviapilosus* sp. nov., holotype, aedeagus. **A** ventral view; **B** dorsal view; **C** lateral view.

Aedeagus (Fig. 3A–C). 1.3 mm long, long and slender, cylindrical. Penis about 1.8 times as long as phallobase; basal half subparallel, then slightly narrowed to basal 0.8, distal 0.2 distinctly cuspidal; endophallus developed reaching distal 0.4 of phallobase; ejaculatory duct with distinct sclerotisation; ventral sac well-developed in distal 1/3, without subapical teeth. Parameres fused to penis, indistinct and apices of parameres reach basal 2/3 of penis.

Measurements. Males: BL 2.9–3.1 mm, BW 1.2–1.3 mm; females: BL 3.0–3.2 mm, BW 1.3–1.4 mm.

#### Diagnosis

This species is different from the other three known species in this genus by elytral intervals 5, 7 and 8 carinate, pronotum and disc of elytra densely pubescent and endophallus more developed reaching distal 0.4 of phallobase.

#### Etymology

The epithet is derived from the Latin adjective “pilosus” = pilous, refers to dense pubescence on disc of elytra.

#### Distribution

China: Guangdong, Guangxi, Jiangxi.

## Discussion

This species also has some characteristics which are different from the three known species in genus *Cuspidevia*, such as the median carinae lacking median sulcus, elytral intervals 5, 7 and 8 carinate and disc of elytra densely pubescent. Carinae on intervals are variable even within the same genus, such as *Grouvellinus*, with carinae on intervals 7, 8 or intervals 5, 7, 8 or intervals 3, 5, 7, 8. Therefore, we thought that the carinae on intervals are not as important as the male genitalia. Endophallus of this new species is quite similar to the three known species of genus *Cuspidevia*. In addition, this new species also has similar characteristics which are similar to the genus *Cuspidevia*, such as anterior angles of pronotum strongly acuminately produced anteriad, elytra broadest near the basal half, elytra striae obsolete in distal 0.6, apices of elytra densely granulate and slightly produced. After comparing with all known species in *Cuspidevia* and *Zaitzevia*, we assigned this new species to the genus *Cuspidevia* (Jiang and Wang 2020, Jiang and Wang 2021, Bian and Zhang 2022, Iwata et al. 2022, Jiang and Chen 2023).

## Supplementary Material

XML Treatment for Cuspidevia pilosus
